# The Utility of a Y-tandem Applicator in a Patient With Stage IVA Locally Advanced Cervical Cancer in the Setting of a Bicornuate Uterus

**DOI:** 10.7759/cureus.77151

**Published:** 2025-01-08

**Authors:** Young Suk Kwon, Himahansika A Weerasinghe, Allen Yen, Astrid Medrano, Brian Hrycushko, Zohaib Iqbal, Kevin Albuquerque

**Affiliations:** 1 Department of Radiation Oncology, The University of Texas, Southwestern Medical Center, Dallas, USA

**Keywords:** brachytherapy, combination brachytherapy, gynec oncology, high dose rate interstitial brachytherapy, locally advanced cervical cancer

## Abstract

We report a case of a patient with a bicornuate unicollis uterus who developed International Federation of Gynecology and Obstetrics (FIGO) Stage IVA adenocarcinoma of the cervix invading the bladder neck, proximal urethra, upper vagina, and parametrium. The patient was treated with definitive chemoradiation therapy, including external beam radiation therapy (EBRT), brachytherapy, and weekly cisplatin infusions. Our patient required an interstitial brachytherapy boost, which was challenging to deliver due to her congenital anatomic anomaly. We were able to utilize the Syed-Neblett template to treat bilateral uteri using the holes in the template base as the entry points for brachytherapy catheters. In addition, a Y-tandem applicator was used to achieve the required angulation at the level of the cervix to reach both uterine walls. After completing both EBRT and brachytherapy, she was free of signs of disease on a follow-up visit approximately two months after treatment. The patient did not have any signs of locoregional disease six months later when she ultimately expired due to widespread pulmonary metastases.

## Introduction

Bicornuate unicollis uterus is a congenital malformation of the uterus with a single vaginal cavity leading to one cervix and two uterine cavities [1]. The lack of brachytherapy guidelines in patients with uterine anomalies may pose challenges in treatment planning [2]. Atypical uterine anatomy may severely compromise the quality of brachytherapy if this is not appropriately recognized ahead of time [3]. Our patient described in this report had locally advanced cervical cancer in the bicornuate uterus. Due to the tumor’s large size and disease infiltration in the surrounding organ, multimodal interventions with chemoradiation were required to ensure optimal oncologic outcomes [4]. In terms of radiation treatment, interstitial brachytherapy is required by patients whose disease cannot be adequately managed by an intracavitary applicator alone due to disease involvement in the vagina and atypical pelvic anatomy resulting in the poor fitting of the applicator [5]. Using the Syed-Neblett template, we were able to use the template base to guide brachytherapy catheters. In addition, we were able to adjust a Y-tandem applicator to cover bilateral uterine walls, although this applicator is typically used for endometrial cancer treatment for covering larger uterine widths [6]. Lastly, mini ovoids were used to stabilize the Y-tandem applicator. With our novel hybrid brachytherapy approach combining intracavitary and interstitial methods along with the use of the Y-tandem applicator, we were able to deliver an interstitial boost of 25 Gy over five fractions using high-dose-rate (HDR) brachytherapy in a patient with a bicornuate uterus, allowing for a cumulative dose of about 80 to the high-risk cervical clinical target volume (CTV) (D90 EQD2 high-risk cervical CTV 79 Gy).

## Case presentation

A 50-year-old African American woman presented to the emergency department (ED), complaining of brownish vaginal discharge. Given the right-sided tenderness in the pelvic exam and leukocytosis, the differential diagnoses at the time included tubo-ovarian/pelvic abscess, pelvic inflammatory disease, and pelvic malignancies. Because she refused hospital admission for an imaging workup, she was discharged with oral antibiotics with the plan of further outpatient diagnostic investigation of the mass.

**Figure 1:**
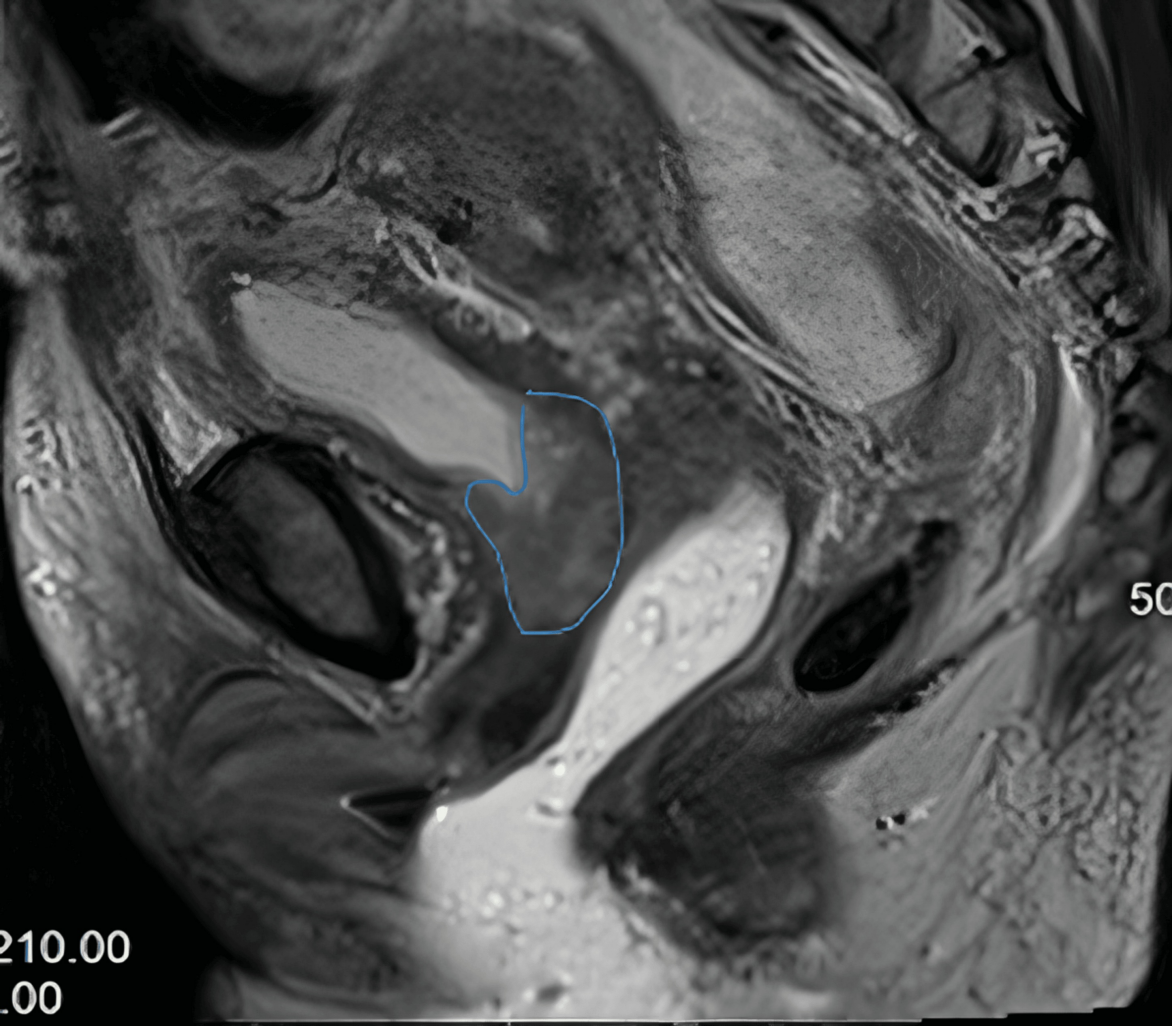
MR T2 sagittal view T2 sagittal MR at diagnosis showing cervical tumor.

**Figure 2:**
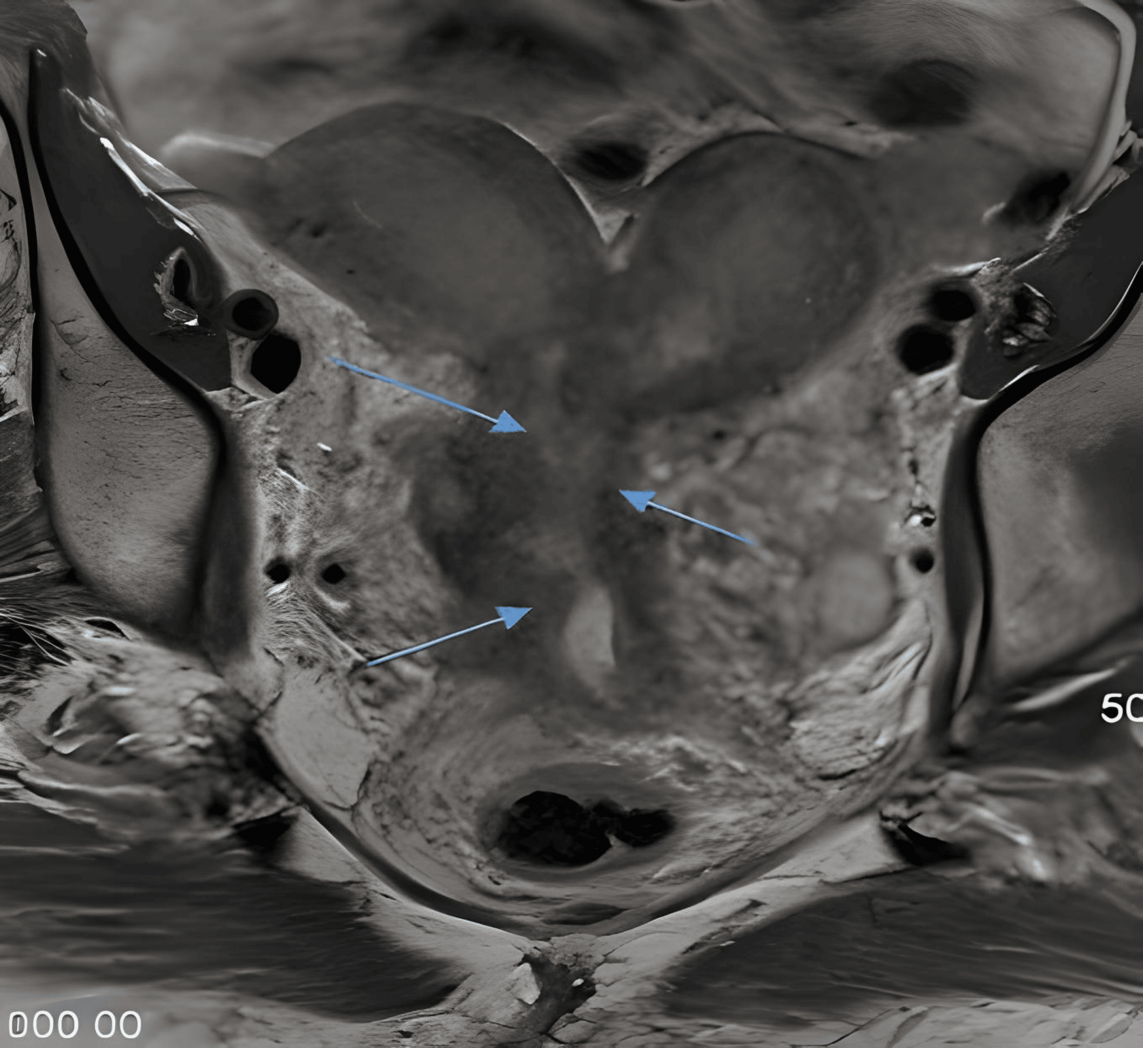
MR T2 coronal view Coronal MRI at diagnosis showing cervical tumor (blue arrows) invading bladder neck, upper vagina, and upper urethra and bicornuate uterus.

A CT urogram performed one month after the ED visit demonstrated an irregular, enhancing soft tissue mass measuring 4.0 x 2.7 x 4.1 cm located in the posterior right abdomen at the L4-L5 level, which abutted the posterior bladder. An MRI of the abdomen confirmed an ill-defined enhancing soft tissue anterior to the right psoas muscle with an associated peripherally enhancing tubular fluid-filled structure that extended to the right lower abdomen in the setting of the bicornuate uterus with a heterogeneously enhancing left uterine horn (Figures 1, 2). A chest CT was unremarkable other than sub-centimeter bilateral pulmonary nodules.

Our patient underwent an examination under anesthesia and cystoscopy four months after the ED visit. Multiple biopsies were taken from the vagina and were positive for adenocarcinoma infiltrating the fibrous stroma. A bimanual pelvic examination demonstrated thick and firm tissue, primarily involving the right vaginal side wall, extending into the right parametrium, starting 4 cm proximal to the hymenal ring. The rectovaginal exam showed fullness involving the right parametrium. A speculum exam revealed obliterated cervical architecture as well as obliterated vaginal fornices and was not able to distinguish cervical from vaginal tissue. Irregular thickened tissue was seen circumferentially, starting 3-4 cm proximal to the hymen with central tunneling, leading to a presumed cervical canal. Endocervical curetting taken from this region was also positive for adenocarcinoma.

A repeat pelvic MRI performed four months after the initial MRI confirmed the presence of a known cervical tumor that invaded the bladder neck, upper vagina, and upper urethra (Figures 1, 2). The right parametrial extension of the tumor was inseparable from components of a complex cystic/tubular and soft tissue process in the right retroperitoneum. The MRI also revealed a bicornuate uterus with findings of adenomyosis.

When the patient presented at our radiation oncology clinic four to five months after the initial presentation, she had worsening back and pelvic pain with more vaginal discharge and bleeding when compared to her initial presentation. On inspection, she presented with normal female external genitalia and a narrow introitus. There was a circumferential lesion 4 cm from the introitus, bulkier on the right, with parametrial involvement, consistent with the prior exam.

Radiation therapy

Our patient was treated with concurrent chemoradiation. She received external beam radiation therapy (EBRT) and interstitial brachytherapy due to bladder involvement and unfavorable anatomy and a stenotic vagina in the setting of a bicornuate uterus. Four cycles of weekly cisplatin (40 mg/m2) were also infused concurrently.

External Beam Radiation Therapy (EBRT)

**Figure 3:**
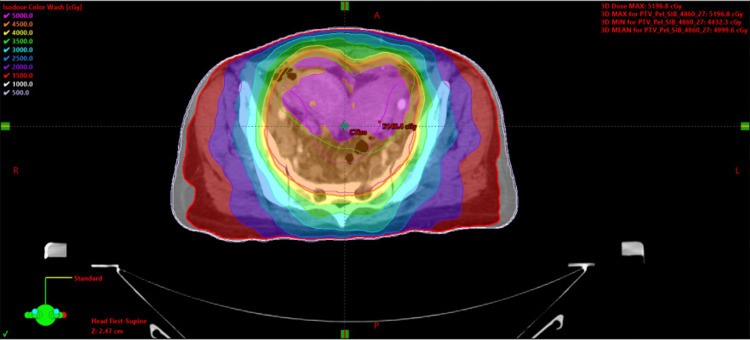
Patient treatment plan in axial view Patient treatment plan axial prescribed to 48.6 Gy over 27 fractions. Dose color wash values are 50 Gy (pink), 45 Gy (orange), 40 Gy (yellow), 35 Gy (green), 30 Gy (light blue), 25 Gy (blue), 20 Gy (purple), 15 Gy (red), 10 Gy (white), and 5 Gy (lavender).

**Figure 4:**
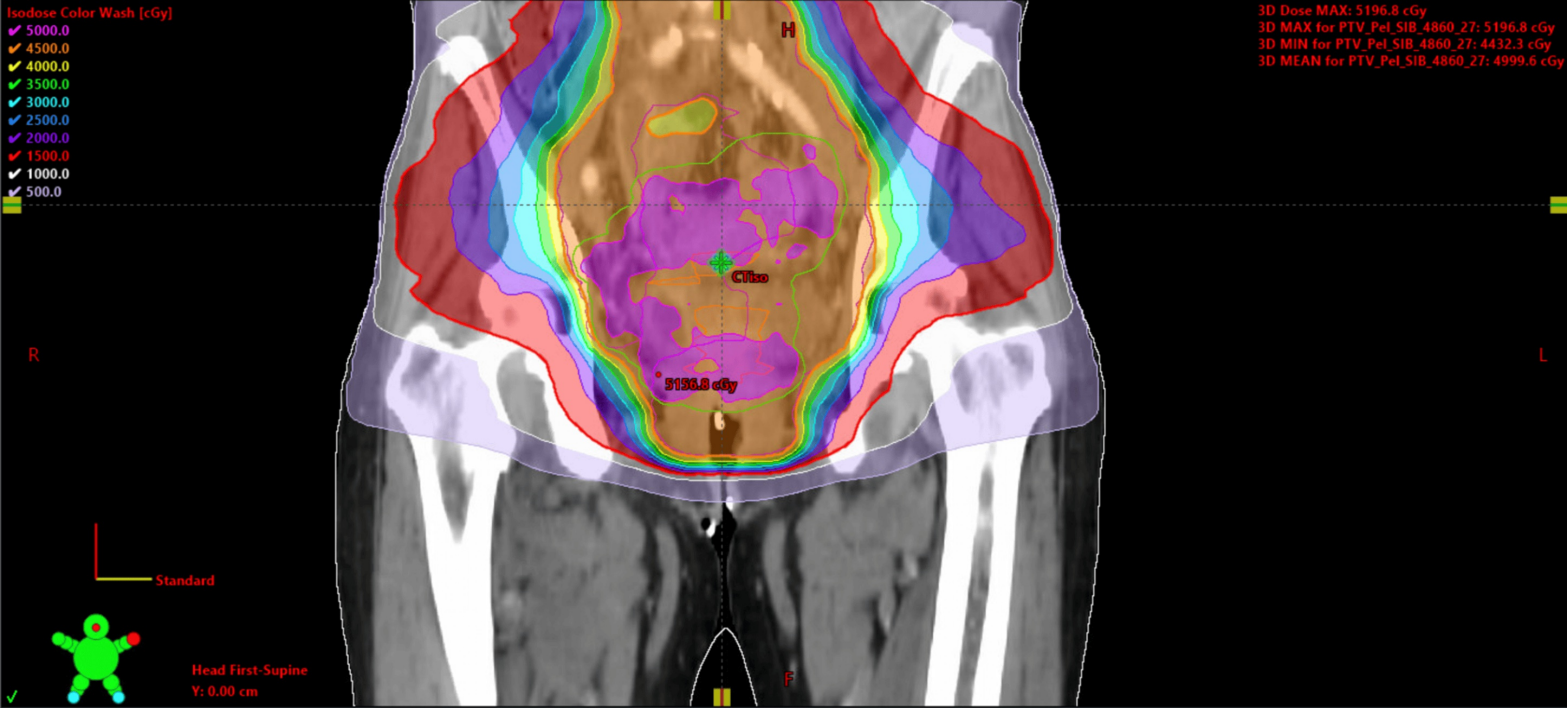
Patient treatment plan in coronal view Patient treatment plan coronal prescribed to 48.6 Gy over 27 fractions. Dose color wash values are 50 Gy (pink), 45 Gy (orange), 40 Gy (yellow), 35 Gy (green), 30 Gy (light blue), 25 Gy (blue), 20 Gy (purple), 15 Gy (red), 10 Gy (white), and 5 Gy (lavender).

**Figure 5:**
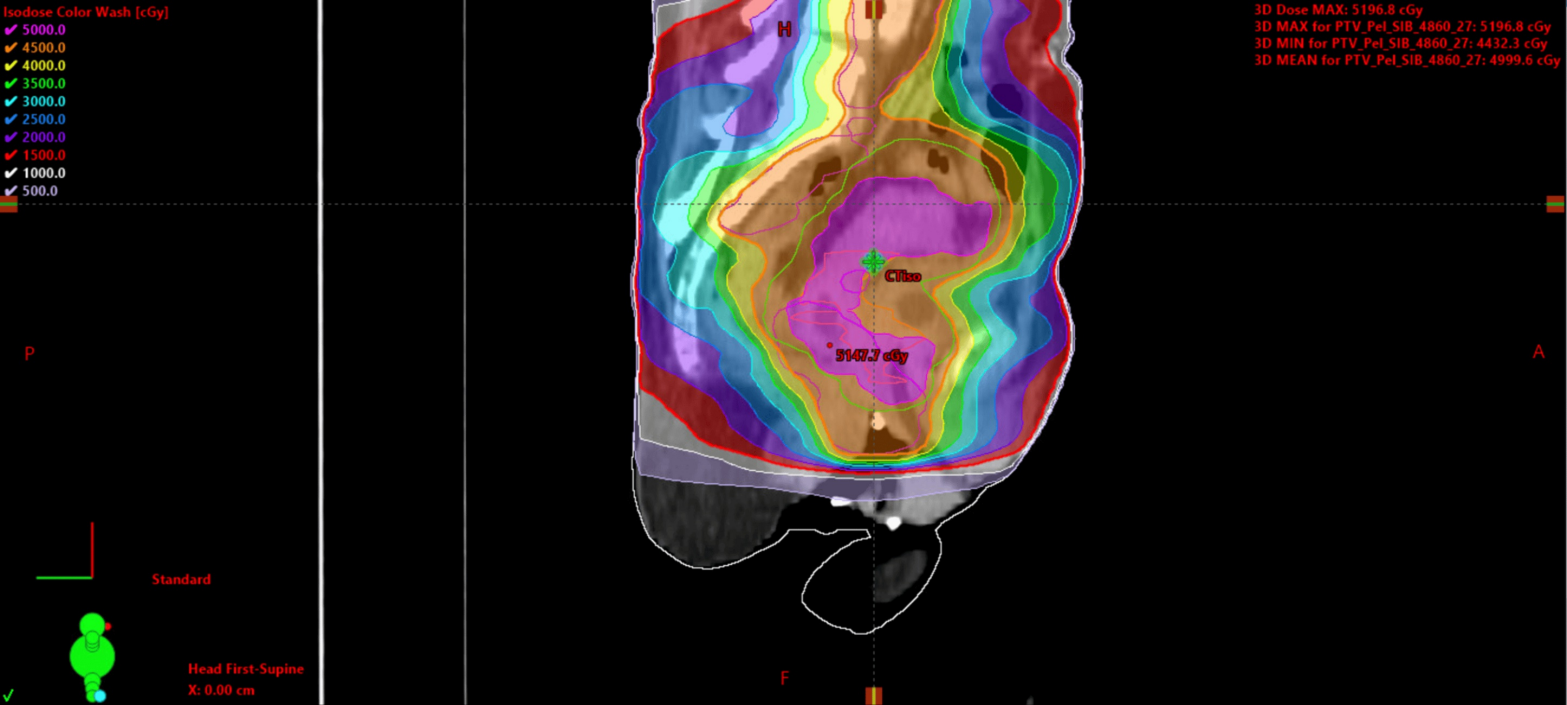
Patient treatment plan in sagittal view Patient treatment plan sagittal prescribed to 48.6 Gy over 27 fractions. Dose color wash values are 50 Gy (pink), 45 Gy (orange), 40 Gy (yellow), 35 Gy (green), 30 Gy (light blue), 25 Gy (blue), 20 Gy (purple), 15 Gy (red), 10 Gy (white), and 5 Gy (lavender).

Our patient received 4860 cGy over 27 fractions using intensity-modulated radiation therapy (IMRT). Multiple clinical target volumes (CTVs), including the cervix CTV, the parametria CTV, the elective nodal CTV, and the vaginal CTV, were constructed for the simultaneous integrated boost technique. A 0.5-cm uniform margin was given for each CTV expansion to a planning target volume (PTV) to reduce bowel toxicity with the image guidance with the CBCT. The CTV vagina, which was expanded with a 1.0 cm margin (Figures 3, 4, 5). The treatment was completed in 43 days due to missed treatments. The patient had multiple ED visits due to hypokalemia during cycle 3 of cisplatin and acute urinary retention requiring a temporary indwelling urinary catheter.

Brachytherapy

The cervical tumor was subsequently treated with HDR interstitial brachytherapy over two days as an inpatient in early March 2021. A total dose of 25 Gy was delivered over five fractions using Iridium-192 over the course of three days (two treatments per day for the first two days and one on the last day). CT and MRI scans were done to accurately delineate parts of radiation treatment and minimize the dose to nearby organs at risk, which included the bowel, bladder, and rectum.

Four fiducial markers were served as radio-opaque markers to establish the four quadrants of the target area. Pelvic examination under anesthesia showed a nodular crater replacing the cervical os. There was disease involvement of the anterior vagina as well as the posterior wall of the bladder and the bladder neck. Given the extensive tumor burden, standard tandem and ovoid were confirmed to be insufficient. Given the unique patient anatomy, no standard templates for interstitial needles would work in this situation. Therefore, based upon the pre-plan prepared by physics staff, a 3D-printed half-template was utilized in order to guide the needle insertion.

**Figure 6:**
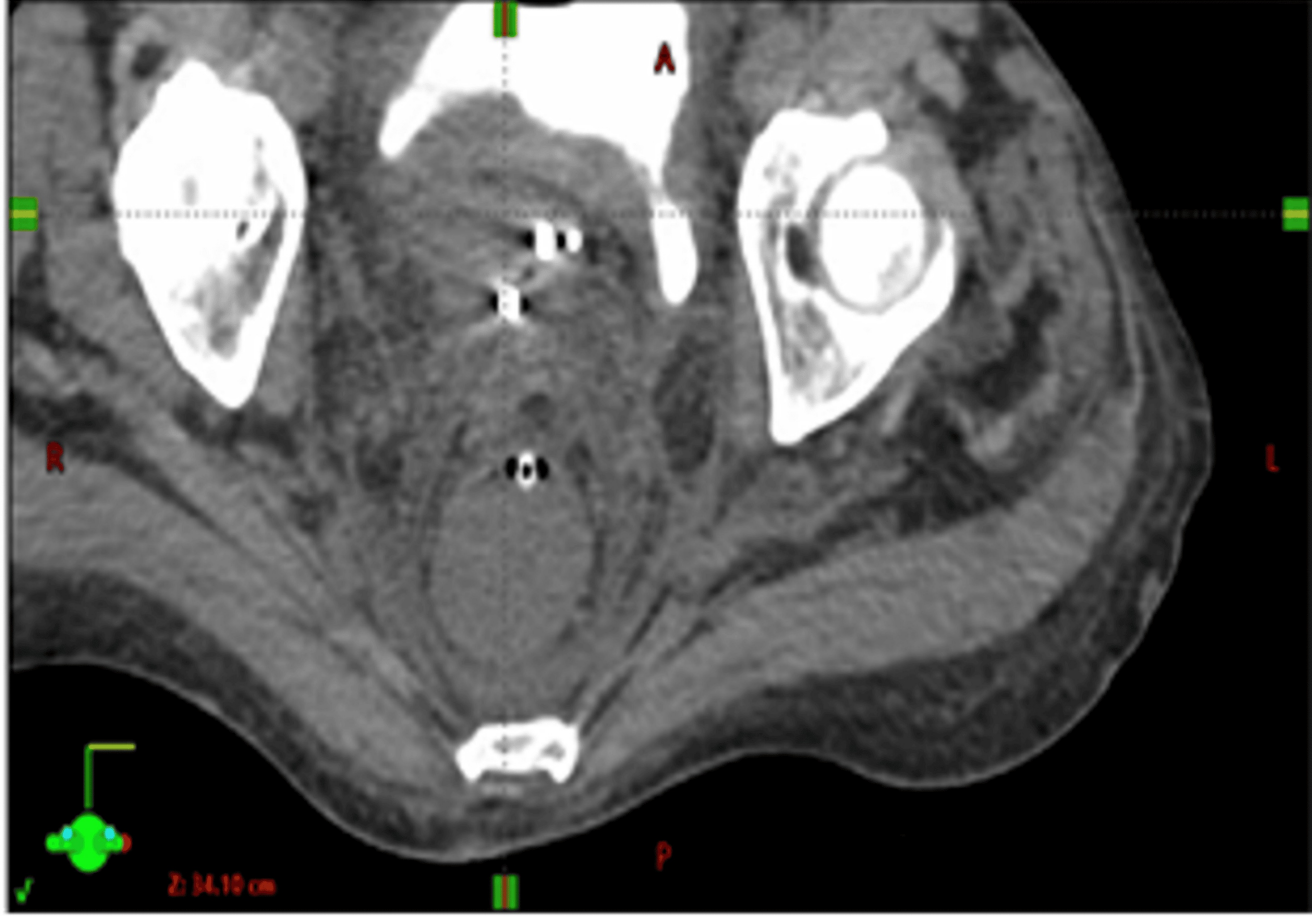
Coronal CT showing mini-ovoids

**Figure 7:**
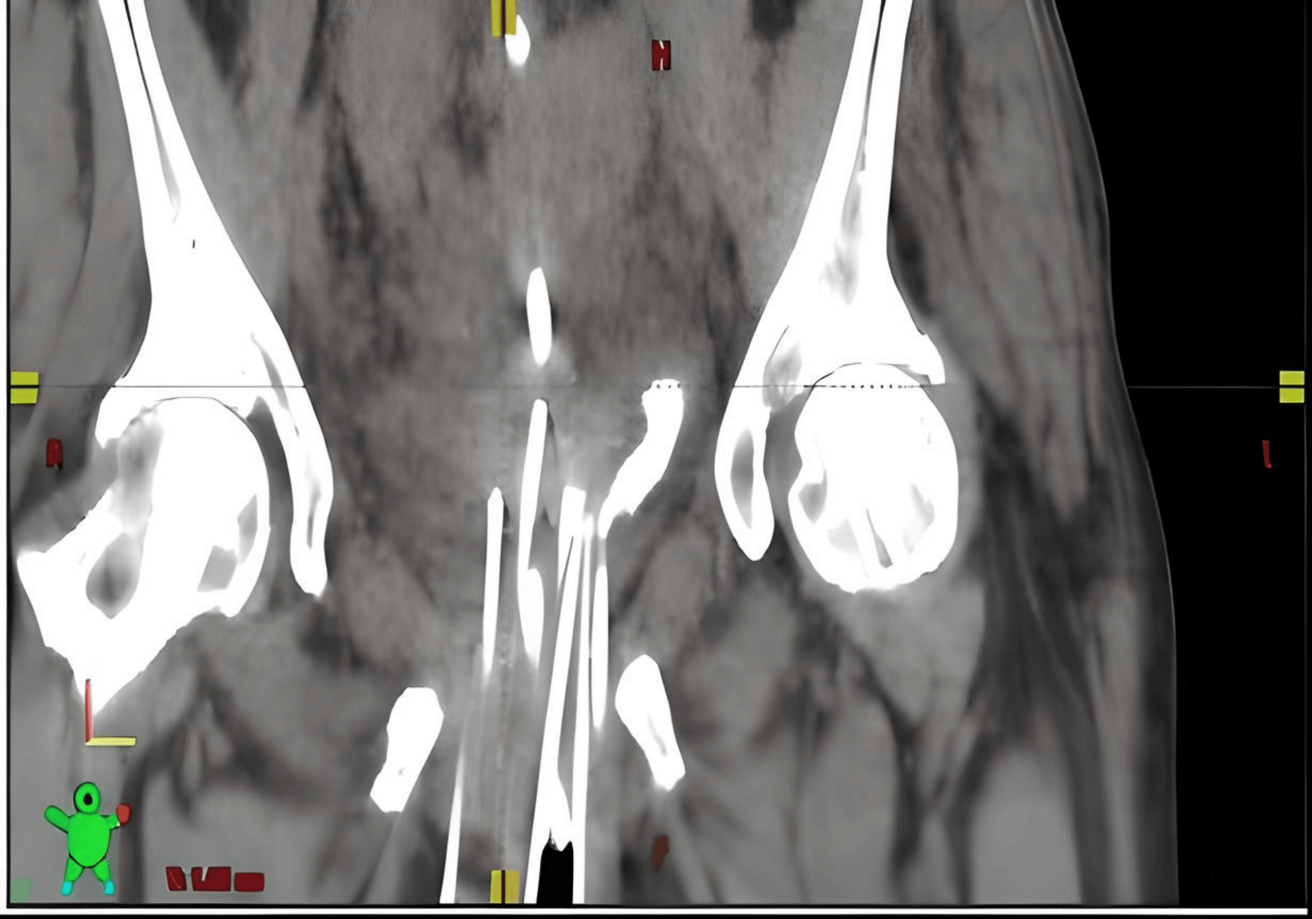
Coronal CT showing Y-tandem

The 3D-printed half-template was placed over the vulva anteriorly with an Ethilon suture, and we used Mepilex wound dressing to protect the skin. Then, under ultrasound guidance, nine needle implants were placed into the periurethral region/bladder neck per our preplan. The implant needles were locked into position using securing screws. Next, the bilateral uteri were sounded on the left and right with ultrasound to approximately 10 cm on both sides. We subsequently placed 8 cm Y-tandems in both uteri and locked them together with the clamp. Because they were not symmetrical and were not able to accommodate regular cylinders, mini-ovoids were placed on both sides to stabilize the Y-tandems and to spread the vaginal canal (Figures 6, 7). A digital rectal exam was performed to confirm the absence of accidental rectal injury. The patient was then cleaned, extubated, and transferred to the post-anesthesia area unit (PACU) uneventfully.

**Figure 8:**
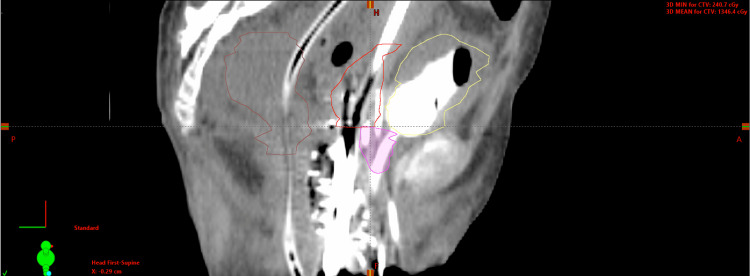
Sagittal CT showing HR-CTV (red) and CTV vagina (purple) The bladder and the rectum were contoured in yellow and brown, respectively.

**Figure 9:**
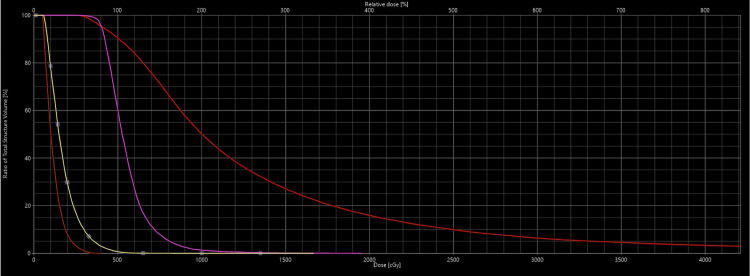
Dose-volume histogram showing the rectum (brown), bladder (yellow), CTV vagina (purple), and HR-CTV (red) (from left to right)

The definition of HR-CTV included gross tumor volume (GTV), the whole cervix, and residual disease. CTV vagina was separately contoured to delineate the extra-cervical tumor extension (Figure 8). For the first fraction, CTV D90% [Gy] was 5.08 Gy (total EQD2 = 79.21 Gy). D2cc of the bladder and rectum were 4.43 Gy and 2.39 Gy (Figure 9).

Follow-up

The patient presented at our clinic for a two-month follow-up. There was no evidence of disease, and she reported good appetite, energy levels back to normal, good urine output, and regular bowel movements. However, she continued to smoke half a pack of cigarettes per day. The patient reported taking ibuprofen or acetaminophen with codeine for occasional pelvic pain that would arise approximately once a week.

Unfortunately, the patient had worsening dyspnea and developed a large bowel obstruction due to a large metastatic implant causing high-grade colonic obstruction at the mid sigmoid colon six months after the follow-up visit, requiring exploratory laparotomy for end-ileostomy and cecostomy. Dyspnea continued to worsen during her hospital stay, with imaging confirming widespread pulmonary metastases, requiring ICU care and intubation. The patient coded several times and was changed to a chemical-only code by the family. She expired on the next code.

## Discussion

Locally advanced cervical cancer in the setting of bicornuate uteri poses both a diagnostic and therapeutic challenge because such anatomic anomalies are not commonly encountered by clinicians. Our case highlights an initial delay with the correct diagnosis partly due to her anatomic anomaly, but more importantly, our experience demonstrates the combination of both intracavitary and interstitial devices to navigate a challenging anatomy associated with bicornuate uteri for the brachytherapy portion of radiation treatment. In our article, we were able to demonstrate the use of the Y-applicator, a device traditionally used for inoperative uterine cancer, for a patient with bicornuate uteri. In addition, we used the Syed-Neblett template interstitial implants to reach bilateral uteri effectively [7]. 

As illustrated in our case, the management of locally advanced cervical cancer in patients with uterine malformations requires individualized therapy. Loo et al. described their experience performing intracavitary brachytherapy with a 3 cm vaginal cylinder for a patient with stage IIB invasive carcinoma of the cervix in the setting of a bicornuate bicollis uterus who was treated with concurrent chemoradiotherapy using EBRT and brachytherapy boost along with weekly cisplatin infusions. Interestingly, the intrauterine catheter was placed on the right uterine canal for fraction 1 and on the left uterine canal for fraction 2. By maintaining the geometry, a total dose of 18 Gy was delivered for both A points [8]. The patient was free of disease on follow-up two years after treatment.

In contrast to the case above, our patient’s anatomy could not allow regular ovoid placement due to a very narrow vagina, ulceration of the cervix seen at the apex, and narrow obliterated fornices. Therefore, we performed interstitial brachytherapy (ICBT) using the Syed-Neblett template with one Y-applicator placed in each uterus. Mini-ovoids were used to secure the Y-tandem. Similar to our experience, there are some reports of combination brachytherapy approaches using both intracavitary and interstitial modalities for patients with bicornuate uteri. We believe that our hybrid approach was an appropriate variation in the brachytherapy technique that could potentially lessen the number of required interstitial needles. 

A case presented by Fabian et al. describes the use of hybrid intracavitary and interstitial applicators to treat a patient with locally advanced cervical cancer in the setting of a bicornuate uterus. They used a tandem, ring applicator, and an interstitial needle and alternated between cornua with each fraction with the interstitial needle placed on opposite sides of the tandem [9]. The local control was excellent, as the patient was disease-free on follow-up 18 months after treatment.

Similarly, Lei et al. presented a study on six patients who received IMRT with ICBT (2D/3D) for locally advanced cervical cancer (stage IIB-IIIB) with uterine didelphys. They used multichannel applicators with one tandem and one flexible intrauterine catheter on each side of the uterine cavity combined with two ovoids placed in the vaginal fornices and/or interstitial needles inserted in large tumors [10]. Four out of the six patients had simultaneous catheterization of the bilateral uterine cavity, and two of them had catheterization of the lesion side. On follow-up, which ranged from eight to 34 months, four patients had complete clinical remission with no recurrence.

## Conclusions

In conclusion, brachytherapy for locally advanced cervical cancer in the setting of uterine anomalies requires individualized treatment, taking into consideration strategies to achieve effective radiation treatment delivery. By ensuring careful applicator placement to deliver the adequate dosage using our innovative aforementioned hybrid techniques combining intracavitary and interstitial approaches, we were able to achieve adequate local control of the disease temporarily, albeit eventual demise. Further investigations are required to understand the optimal workflow, including physical exams, imaging workup, and radiation preplan, in order to generate brachytherapy techniques for patients with atypical anatomy due to disease processes as well as congenital anomalies.
